# Structural Basis of Microtubule Destabilization by Potent Auristatin Anti-Mitotics

**DOI:** 10.1371/journal.pone.0160890

**Published:** 2016-08-12

**Authors:** Andrew B. Waight, Katja Bargsten, Svetlana Doronina, Michel O. Steinmetz, Django Sussman, Andrea E. Prota

**Affiliations:** 1 Department of Protein Sciences, Seattle Genetics, Inc., Bothell, WA, United States of America; 2 Department of Biology and Chemistry, Laboratory of Biomolecular Research, Paul Scherrer Institut, Villigen PSI, Switzerland; University of Illinois at Chicago, UNITED STATES

## Abstract

The auristatin class of microtubule destabilizers are highly potent cytotoxic agents against several cancer cell types when delivered as antibody drug conjugates. Here we describe the high resolution structures of tubulin in complex with both monomethyl auristatin E and F and unambiguously define the *trans*-configuration of both ligands at the Val-Dil amide bond in their tubulin bound state. Moreover, we illustrate how peptidic vinca-site agents carrying terminal carboxylate residues may exploit an observed extended hydrogen bond network with the M-loop Arg278 to greatly improve the affinity of the corresponding analogs and to maintain the M-loop in an incompatible conformation for productive lateral tubulin-tubulin contacts in microtubules. Our results highlight a potential, previously undescribed molecular mechanism by which peptidic vinca-site agents maintain unparalleled potency as microtubule-destabilizing agents.

## Introduction

Microtubules are highly dynamic cytoskeletal protein polymers composed of repeating αβ-tubulin heterodimers, and are essential for multiple cellular processes in eukaryotes including cell division, differentiation, transport and motility. Microtubule dynamic instability arises via the regulated association and disassociation events of individual tubulin dimers from microtubule ends and is linked to GTP hydrolysis [1,2]. The non-equilibrium polymerization of microtubules can be abruptly reversed by a disassembly process known as catastrophe, which releases GDP-tubulin [3]. The exchange of GDP to GTP regenerates the polymerizable form of tubulin, and therefore plays an important role in the cellular regulation of microtubule dynamics [4].

The tubulin dimers of the microtubule cytoskeleton are the cellular target for a substantial number of naturally occurring cytotoxic agents that are believed to act primarily by the disruption of mitosis [5], and by interfering with interphase microtubule function, such as trafficking that is essential for cell metabolism and/or signaling [6]. These molecules are loosely classified by the general regions on the tubulin dimer to which they bind. The tubulin binding agents which bind at or near to sites of the vinca alkaloids are referred to as vinca-site antimitotics [7]. Vinca-site binding compounds originate from disparate organisms and exhibit a variety of chemical compositions [8]. Several of these compounds, such as vincristine and vinblastine, have been used for more than four decades as clinically effective cancer therapeutics [7]. More recently, highly potent antimitotics such as maytansine and monomethyl auristatin E (MMAE) have been successfully implemented as cytotoxic payloads for antibody-drug conjugates (ADC) [9–11]. These advances have resulted in newly approved cancer therapeutics with outstanding efficacy.

**Fig 1:**
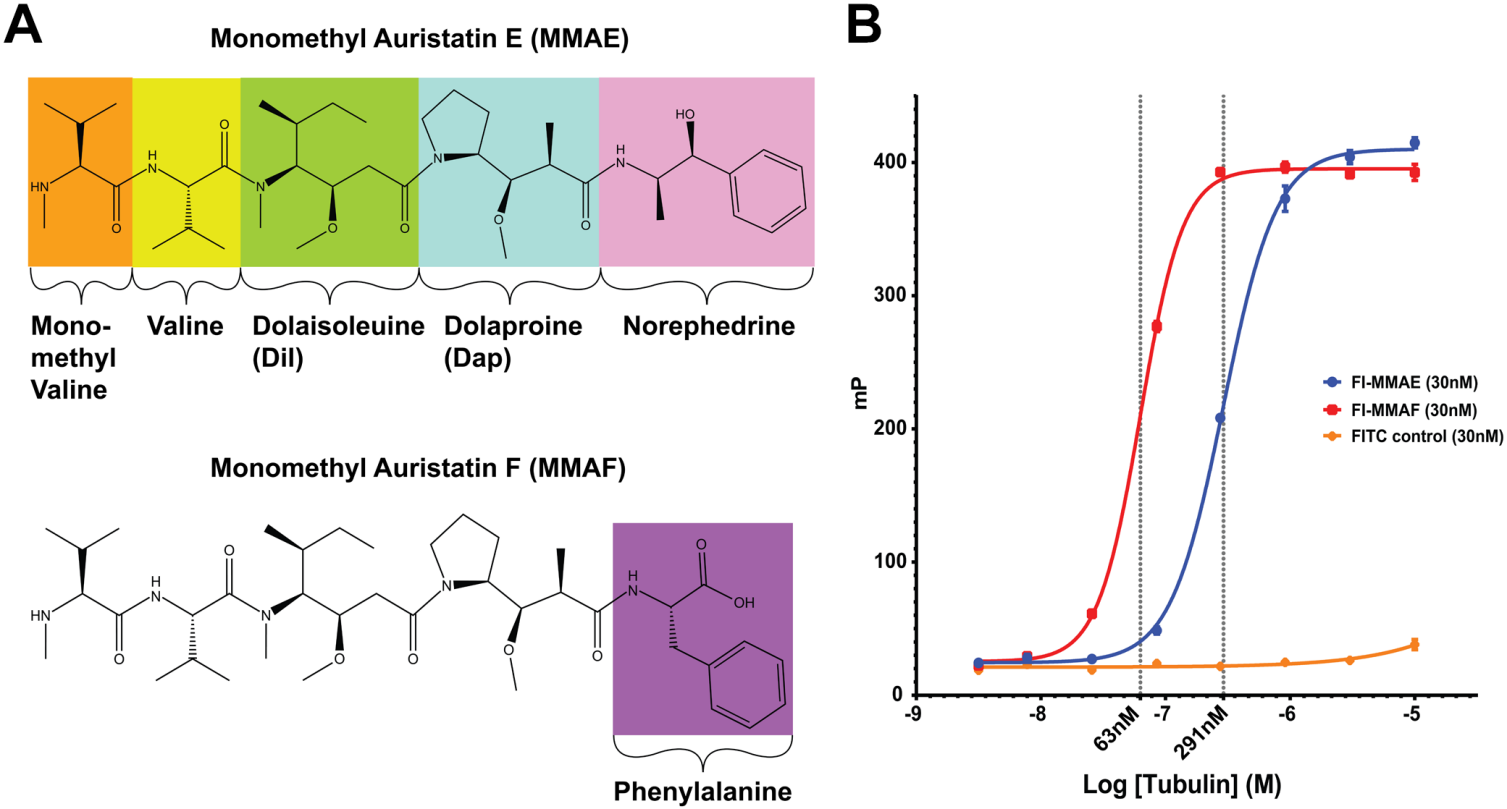
Chemical structures and tubulin binding characteristics of compounds used in this study. **(A)** Amino acid constitutents of monomethyl auristatin E (MMAE) and monomethyl auristatin F (MMAF). The four amino acids of MMAE are labeled and highlighted in color. The carboxy-terminal difference between the norephedrine group of MMAE and the phenylalanine MMAF is highlighted in pink and violetpurple, respectively. **(B)** Fluorescence polarization binding assay of FITC derivatives of MMAE (blue) and MMAF (red) to free tubulin. FITC-conjugated butylamine (orange) was used as negative control. Assay is conducted in 20 mM PIPES buffer pH 6.9 and 1 mM MgCl_2_. Ordinate values are arbitrary polarization units (P) and the abscissa denotes the log molar concentration of sheep brain tubulin. Data points are mean values from triplicate experiments with error bars representing standard deviations. K_D_ values are 291 nM for FI-MMAE and 63 nM for FI-MMAF.

MMAE is a highly potent synthetic analog of the natural peptide product dolastatin 10 [9,12,13]. The mechanisms by which these molecules act on tubulin dimers include the induction of curved aggregates and inhibition of nucleotide exchange [14]. MMAE comprises the four amino acids monomethylvaline (MeVal), valine (Val), dolaisoleuine (Dil) and dolaproine (Dap), and the carboxy-terminal amine norephedrine (Fig 1A). This agent provides the cytotoxic activity for the ADC Brentuximab vedotin, currently designated for relapsed Hodgkin lymphoma and anaplastic large cell lymphoma [11]. Brentuximab vedotin consists of approximately four MMAE molecules conjugated via a protease-cleavable linker to an anti-CD30 antibody. Upon binding to cells expressing the CD30 epitope, the ADC is internalized and MMAE is released into the cytosol resulting in mitotic arrest and apoptosis [9]. The cleaved and released MMAE molecule is indistinguishable from the freely synthesized compound.

Crystal structures of vinca-site ligands in complex with tubulin show that the binding region of these compounds is located at the interface between two longitudinally aligned tubulin dimers, between a β_1_-tubulin subunit and the adjacent α_2_-tubulin subunit [15,16]. The positioning of vinca-site compounds in this region suggests that these molecules maintain the two longitudinally aligned tubulin dimers in a curved conformation that is incompatible with the straight structure of microtubules. This model of curvature between dimers is supported *in vitro* by the formation of curved spiral or ring shaped aggregates that are, for example, induced by the vinca alkaloids vinblastine, cryptophycin and dolastatin [8].

Biophysical and biochemical experiments with free tubulin and dolastatin 10 have suggested that this compound is a non-competitive inhibitor of the vinca alkaloids and shows a much greater inhibition of nucleotide exchange [17]. These observations were the first demonstration that the members of the auristatin class of compounds interact with a structurally distinct region from the vinca site, termed the peptide site [17]. Low resolution crystal structures of dolastatin 10 derivatives in complex with tubulin have further confirmed the region of binding for the auristatins, which overlaps with that of the vinca site but extends significantly further to interact with the bound GDP ligand at the exchangeable site on β-tubulin [18,19]. In addition, recently reported moderate resolution (between 3.1 and 3.5 Ǻ) crystal structures of dolastatin 10 analogs have described the occurrence of a *cis*-configuration at the Val-Dil amide bond in their bound state to tubulin, which is in contrast to the *trans*-configuration observed in solution [19]. However, at such low resolutions, the electron density is not detailed enough to unambiguously distinguish between the two possible configurations of the ligand in the binding site.

To enhance our understanding of how auristatins bind to the peptide site and to elucidate the existing chemical structure-activity relationships of auristatin analogs [12], we have determined the crystal structures of both MMAE and monomethyl auristatin F (MMAF) in complex with tubulin (MMA-structures) to 1.8 and 2.5 Ǻ resolution, respectively. Analysis of these structures allows for the unambiguous determination of the *trans*-configuration at the Val-Dil amide bond in the bound state. Moreover, comparison of the MMA-structures and other tubulin structures liganded to vinca-site ligands, including a vinblastine complex in the same crystal form as the MMAs, reveals a cross-talk to the M-loop through Arg278, thereby suggesting an additional general mechanism of microtubule destabilization by vinca-site ligands. Understanding the atomic mechanisms responsible for the potency of MMAE and its derivatives will greatly aid in the development of new antimitotic compounds with auristatin-like properties.

## Materials and Methods

### Fluorescence polarization binding assay

Sheep brain tubulin was obtained from Cytoskeleton (Cytoskeleton Inc Denver, CO) and exact protein concentration was determined using the DC protein assay (Bio-Rad Laboratories, Hercules, CA) 8 point serial dilutions of tubulin were conducted using FP assay buffer (20 mM PIPES pH 6.9, 1 mM EGTA, 1 mM MgCl_2_) at 2X assay concentration (highest amount 20 μM and dilutions occurring at 3.3X concentration). Starting with a 1 mM FITC labeled auristatin stock solution in DMSO probe was diluted to 60 nM in FP assay buffer + 0.008% Tween 20. To initiate the assay, 15 μL of 2X tubulin serial dilution was combined with 15 μL 60 nm FITC labeled stock in the wells of a 384 well plate (Corning #3575) for a final concentration of 30 nM fluorophore and 8 tubulin concentration points (10000.00, 3030.30, 918.27, 278.26, 84.32, 25.55, 7.74, and 0.00 nM) performed in triplicate. The plate was covered with aluminum foil and the reaction was allowed to proceed for 1 hour at room temperature with gentle shaking. Fluorescence polarization was measured on an Envision multilabel reader (Perkin Elmer, Waltham, MA) using an installed FITC FP dual mirror (#2100–4070). Measurements of polarization (milli-polarization units) are defined as (mP) = 1000*(S-G*P)/(S+G*P) where S and P represent the parallel and perpendicular background subtracted fluorescence count rates following polarized excitation, and G (grating) is an instrument dependent factor calculated from pure fluorophore solution. Binding data was analyzed using Graphpad Prism software (GraphPad Software, Inc. La Jolla, CA). Kd values were extrapolated from tubulin concentration EC50 values calculated from a dose-response variable slope model given by the equation [Y = Bottom + (Top-Bottom)/(1+10^((LogEC50-X)*HillSlope))].

### Protein expression and purification

Bovine brain tubulin was prepared according to ref. 20 [20]. Methods for the production of T_2_R-TTL complex using the stathmin-like domain of RB3 and chicken TTL expressed in E. coli, have been described in refs. 21, 22 [21,22] and 23 [23].

### Crystallization, data collection, and structure solution

Crystals of T_2_R-TTL were generated as described previously in refs. 21 [21] and 22 [22]. Suitable T_2_R-TTL crystals were exchanged into reservoir solutions containing either 2 mM MMAE, 1 mM MMAF or 1 mM vinblastine and soaked overnight (MMAE) or for 1 hour, respectively. Soaked crystals were flash cooled in liquid nitrogen following a brief stepwise transfer into cryo solutions containing 15% and 20% glycerol, respectively. T_2_R-TTL MMAE and vinblastine data were collected at beamlines X10SA and X06SA at the Swiss Light Source (Paul Scherrer Institut, Villigen, Switzerland), respectively. T_2_R-TTL MMAF data were collected at data beamline 4.2.2 at the Advanced Light Source (Lawrence Berkeley Labs, Berkeley, USA). Images were indexed and processed using XDS, and structure solution by the difference Fourier method and refinement were performed using the PHENIX package. Model building was carried out iteratively using the Coot software. Data collection and refinement statistics are given in Table 1. The atomic coordinates have been deposited in the Protein Data Bank, www.rcsb.org [PDB ID code 5IYZ (T_2_R-TTL-MMAE), 5J2U (T_2_R-TTL-MMAF), 5J2T (T_2_R-TTL-vinblastine)].

**Table 1:** Data collection and refinement statistics.

	T_2_R-TTL-MMAE	T_2_R-TTL-MMAF	T_2_R-TTL-vinblastine
**Data collection**^a^			
Space group	P2_1_2_1_2_1_	P2_1_2_1_2_1_	P2_1_2_1_2_1_
Cell dimensions			
*a*, *b*, *c* (Å)	104.5, 156.6, 182.4	104.6, 155.4, 182.5	105.3, 157.7, 182.5
Resolution (Å)	71.9–1.80 (1.85–1.80)	56.65–2.5 (2.64–2.50)	48.2–2.2 (2.26–2.20)
R_meas_ (%)	6.1 (267.6)	16.6 (204.7)	16.5 (362.3)
R_pim_ (%)	2.6 (108.8)	6.3 (85.7)	5.0 (127.0)
CC_1/2_^b^	99.9 (28.2)	99.6 (41.1)	99.9 (34.5)
I/σI	17.3 (0.8)	8.7 (0.8)	11.9 (0.7)
Completeness (%)	99.9 (99.5)	99.0 (93.2)	97.8 (92.0)
Redundancy	6.7 (6.2)	6.9 (5.2)	12.9 (11.4)
**Refinement**			
Resolution (Å)	71.9–1.80	56.65–2.5	48.2–2.20
No. unique reflections	275542	102228	150857
R_work_/R_free_ (%)	16.7 / 20.2	21.6 / 25.0	19.3 / 24.4
Average B-factors (Å^2^)			
Complex	54.5	72.3	69.0
Solvent	56.3	56.5	59.9
Ligands (chain B/D)	33.3 / 106.4	48.4 / 107.0	49.9
Wilson B-factor	37.2	55.8	50.1
Root mean square deviation from ideality			
Bond length (Å)	0.009	0.005	0.008
Bond angles (°)	1.157	0.903	1.074
Ramachandran statistics^c^			
Favored regions (%)	98.3	95.3	95.9
Allowed regions (%)	1.7	4.6	3.6
Outliers (%)	0.0	0.1	0.5

^a^Highest shell statistics are in parentheses.

^b^CC_1/2_ = percentage of correlation between intensities from random half-datasets.

^c^As defined by MolProbity.

### Structural analysis and figure preparation

**Fig 2:**
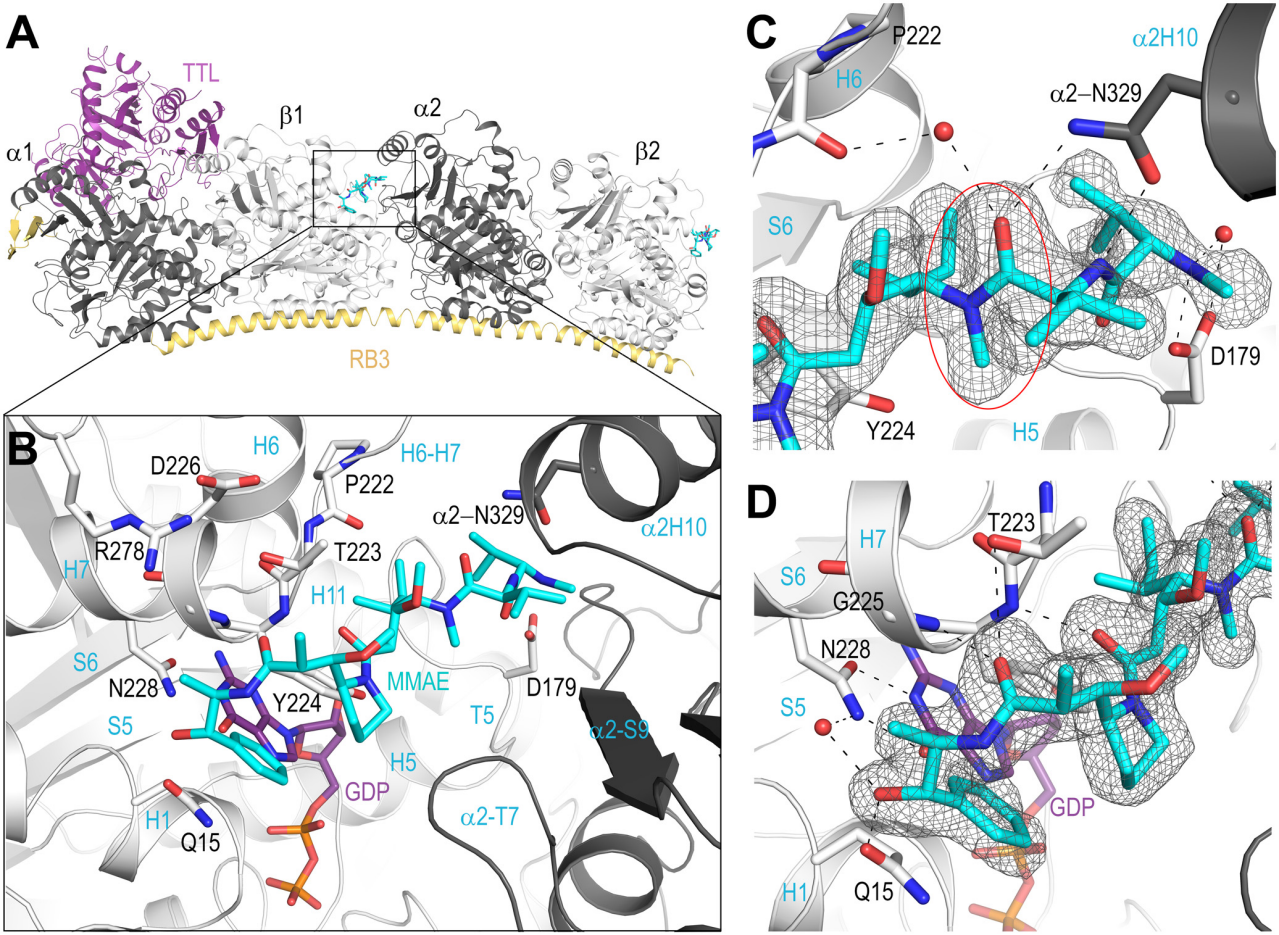
Location of the MMAE binding site in the T_2_R-TTL complex. (**A**) The entire T_2_R-TTL complex is shown with TTL (purple) and RB3 (yelloworange), α (dark gray) and β tubulin (light gray) subunits. MMAE molecules (cyan) are bound to the complex at both the high affinity β_1_/α_2_ interface, and to the lower affinity β_2_ subunit alone, however density for the low affinity site is poorly resolved. (**B**) The high affinity MMAE binding site, colored as in panel A. The MMAE molecule makes the most extensive contacts with the H6-H7 loop on the β subunit, and the carboxy-terminal norephedrine is located directly above the GDP ligand. The amino-terminus of the molecule primarily interacts with the βT5 loop and αH10 on the adjacent subunit. (**C**) Specific interactions of the MMAE amino-terminal residues. Asp179 located on the T5 loop interacts with the positively charged N-methyl group and co-coordinates a crystallographic water. The side chain of Asn329 on αH10 forms a dual interaction with the amide nitrogen and carbonyl group of the MMAE valine residue. A crystallographic water molecule is also located between the MMAE valine carbonyl and the carbonyl of the Pro222 of the βH6-H7 loop. The trans-configuration observed at the Val-Dil amide bond of the bound MMAE is highlighted with a red ellipse. (**D**) Specific interactions of the MMAE carboxy-terminal residues. The carbonyl groups of both Dil and Dap form hydrogen bonds to the amide nitrogens of Tyr224 and Gly225, respectively. The carboxy-terminal norephedrine is positioned directly above the GDP ligand and the hydroxyl group forms interactions with the side chain of Gln15 and coordinates a crystallographic water molecule with Asn228. The SigmaA‐weighted mFo‐DFc omit map (grey mesh) in both the panels **C** and **D** is contoured at + 3.0σ.

Molecular graphics and analyses were performed with the UCSF Chimera package and PyMol (The PyMOL Molecular Graphics System, Version 1.5.0.5. Schrödinger, LLC). Chimera is developed by the Resource for Biocomputing, Visualization, and Informatics at the University of California, San Francisco (supported by NIGMS P41-GM103311). Chains in the T_2_R-TTL complex were defined as follows: chain A, α-tubulin-1; chain B, β-tubulin-1; chain C, α-tubulin-2; chain D, β-tubulin-2; chain E, RB3; and chain F, TTL (Fig 2).

## Results and Discussion

### Determination of binding activities by fluorescence polarization assay

The *in vitro* and *in vivo* properties of MMAE have previously been described in detail both for the free drug and as an ADC [24]. The augmented activity and properties of the carboxy-terminally charged monomethyl auristatin F (MMAF) derivative have likewise been reported for both cleavable and non-cleavable conjugates [25]. However, direct equilibrium dissociation constants, K_D_’s, of auristatins to free tubulin have not been reported. Reliable and facile K_D_ measurements have proved challenging due to both the complexity of the protein system being investigated and the propensity of the auristatin ligands to promote longitudinal aggregation of the tubulin dimers. We have developed a simple and highly reproducible fluorescence polarization assay to ascertain the binding activity of FITC conjugated analogs of MMAE (FI-MMAE) and MMAF (FI-MMAF) and which can be used to evaluate either the K_D_ values of FITC conjugates directly or the apparent IC_50_ values of unlabeled chemotypes in competition assays. Fluorescence polarization binding measurements of FI-MMAE and FI-MMAF to free tubulin demonstrate K_D_ values of 291 and 60 nM (±3 nM), respectively (Fig 1B). These measurements demonstrate nearly a ~5 fold increase in the binding affinity by the replacement of the carboxy-terminal norephedrine moiety of MMAE with the phenylalanine amino acid found in MMAF. These results further suggest that the >100 fold increase in cellular toxicity exhibited by membrane permeable MMAF analogs over MMAE [25] is at least partly a result of enhanced tubulin binding affinity.

### The crystal structure of tubulin in complex with MMAs

To investigate the specific mode of binding as well as differences in activity between MMAE and MMAF, we used a protein complex composed of two αβ-tubulin (T_2_), the stathmin-like protein RB3 (R) and tubulin tyrosine ligase (TTL), and determined the crystal structures of both the liganded auristatin analogs MMAE (T_2_R-TTL-MMAE) and MMAF (T_2_R-TTL-MMAF) at 1.8 and 2.5 Å resolution (Table 1). Furthermore, to compare their binding modes to vinblastine in the same crystal form, we determined the crystal structure of T_2_R-TTL in complex with vinblastine to 2.2 Å resolution (Table 1). The T_2_R-TTL-MMAE and T_2_R-TTL-MMAF structures are nearly identical (RMSD of 0.24 Å over 440 Cα atoms; β_1_-tubulin chain), and are highly similar to the drug-free T_2_R-TTL complex [22] (PDB-ID 4IHJ; 0.26 Å over 440 Cα atoms). The T_2_R-TTL-vinblastine structure is also very similar and superimposes to the β_1_-tubulin chain of T_2_R-TTL-MMAE with an RMSD of 0.50 Å over 375 Cα atoms.

Consistent with other structural reports for peptide based vinca-site binders [18,19], MMAE binds at a distinct and previously suggested peptide site [14] on the β-tubulin subunit at the inter-dimer interface between two longitudinally aligned tubulin molecules (Fig 2A). However, in contrast to vinblastine [15], MMAE also interacts with the exposed β_2_-tubulin subunit of the second tubulin dimer in the T_2_R-TTL complex (Fig 2A). This mode of binding agrees with other structural reports for peptide based vinca-site binders and further confirms a distinct and previously suggested peptide site on the β-tubulin subunit [18,19]. The amino-terminus of MMAE projects into the vinblastine binding site, however, the carboxy-terminal end extends further into the interdimer interface to position the terminal norephedrine group directly above the bound GDP ligand (Fig 2B). As a consequence, and compared to vinblastine, which interacts almost equally across the interface (326 Å^2^ β_1_-tubulin, 359 Å^2^ α_2_-tubulin), MMAE shares a greater buried surface area with the β_1_-tubulin subunit (457 Å^2^) than with the adjacent α_2_-tubulin subunit (273 Å^2^). The MMAE binding site is composed of the β_1_-tubulin T5 loop, the carboxy-terminal end of the H6 helix, the H6-H7 loop, the amino-terminus of the H7 helix and the H1 helix (Fig 2B). The site is completed on the α_2_-tubulin subunit side by the S9 strand, the H10 helix and the T7 loop. Unlike the binding mode of vinblastine, the MMAE molecule is elongated towards the nucleotide binding site, forming more extensive interactions with the β_1_-tubulin subunit H6-H7 loop and H7 helix. This binding mode is more similar to phomopsin and soblidotin; however, unlike these agents, the carboxy-terminus of the MMAE molecule interacts with the H1 helix and rests directly on the purine carbonyl oxygen of the GDP ligand [18]. The members of the auristatin class of molecules have been shown to potently inhibit nucleotide exchange on tubulin without displacement of the bound nucleotide [14], and the interactions proximal to the bound GDP ligand confirm a structural basis for this biochemical property.

### The detailed MMAE binding mode

The high resolution crystal structure of MMAE elucidates the specific interactions that are essential for both the affinity and potency for this class of molecules [12]. Starting with the amino-terminal end of the MMAE molecule, the nitrogen of the methylated valine amino terminus forms a hydrogen bond to one side chain carboxylate oxygen of Asp179, found on the T5 loop of the β_1_-tubulin subunit (Fig 2C). It is interesting to note that the geometry of the methylamine group in this position interacts with only one of the δ oxygen atoms of the Asp179 carboxylate. The interaction with the other oxygen atom occurs through a coordinated crystallographic water molecule. The second residue in the MMAE molecule is a natural amino acid valine, and this side chain projects into a hydrophobic pocket on the adjacent α-tubulin subunit. Importantly, both the amide nitrogen and carbonyl moieties of the MMAE backbone here also interact with the α_2_-tubulin subunit through hydrogen bonds to the Asn329 side chain Oδ1 and Nδ2 atoms, respectively. The same carbonyl additionally co-coordinates a crystallographic water molecule that is shared with the carbonyl of the backbone for Pro222 of the β_1_-tubulin subunit (Fig 2C).

The side chain of the γ-amino acid Dolaisoleuine occupies a hydrophobic pocket located on the β_1_-tubulin subunit and the O-methyl group of this residue orients the subsequent carbonyl towards the formation of a hydrogen bond to the amide backbone of the Tyr224 residue on the β_1_-tubulin subunit (Fig 2D). Likewise, the Dolaproline residue maintains a particular configuration for the positioning of its carboxy-terminal carbonyl to maintain a hydrogen bond with the amide backbone of Gly225. Also in this region, the tyrosyl ring of Tyr224 is shifted 1 Å away from the GDP as compared to the drug free tubulin structure, likely as a result of auristatin binding. The phenyl group of the carboxy-terminal norephedrine residue is perfectly oriented to stabilize the bound GDP ligand. The phenyl ring occupies a groove flanked by Tyr224 and Gln15 of the β_1_-tubulin subunit, and the hydroxyl group forms both a hydrogen bond to the Oε2 of Gln15 and coordinates a water molecule with the Nδ2 of Asn228 (Fig 2D). Importantly, the Gln15 side chain is rotated from the position found in both the drug free tubulin and the tubulin-vinblastine structures, and this adjustment moves the Oε2 atom ~4Å away from a hydrogen bond to the O6 guanosine carbonyl of the bound GDP. Our crystallographic analysis highlights the importance of the unique Dolaisoleuine and Dolaproine γ-amino acids which maintain a precise distance and angular orientation of each respective carbonyl group to form hydrogen bonds to the backbone amides of β_1_-tubulin Tyr224 and Gly225 in the T_2_R-TTL complex. These two hydrogen bonds critically maintain the auristatin association to the β_2_-tubulin subunit that is otherwise solvent exposed in this region, and serve to position the carboxy-terminus in a location ideal for the inhibition of nucleotide exchange (Fig 2B). This association is further stabilized by the *trans*-configuration (next paragraph) at the amide bond between the Valine and Dolaisoleuine constituents, which favorably orients the backbone amide and carbonyl of MMAE to form additional hydrogen bonds to both the backbone carbonyl of Pro222 and the Asn329 side chain of the longitudinally aligned α_2_-tubulin subunit. These observations are in agreement with the SAR-study presented by Pettit and coworkers [12] and with the binding mode that was recently described by Wang and co-workers [26].

### The *trans*-configuration at the Val-Dil amide bond

Interestingly, the reported 3.1 Å crystal structures of dolastatin 10 and amino-terminal auristatin analogs denote a *cis*-configuration between the Valine and Dil components of the bound ligand at this location [19]. Moreover, in these structures the *cis*-configuration is only seen by ligands bound at the β_1_/α_2_ tubulin interface, whereas those bound to the free β_2_-tubulin subunit display the lower energy *trans*-configuration. These observations, along with NMR solution data supporting a *trans*-configuration for free auristatin analogs, has led to the proposal that auristatins and related molecules maintain a *trans*-configuration during initial β_1_-tubulin subunit binding and reconfigure to a *cis*-amide upon subsequent α_2_–tubulin subunit addition. Although not discussed by Maderna and co-workers [19], it is also possible that *trans-*/*cis-*switching occurred under soaking conditions and that the *cis-*conformer of the peptide was crystallized. In contrast to these previous observations, both the T_2_R-TTL-MMAE and T_2_R-TTL-MMAF structures unambiguously display a *trans*-configuration for the amide bond between the Valine and Dil constituents of the bound ligands at the β_1_/α_2_ tubulin interface (Fig 2B and 2C).

### The detailed MMAF binding mode

**Fig 3:**
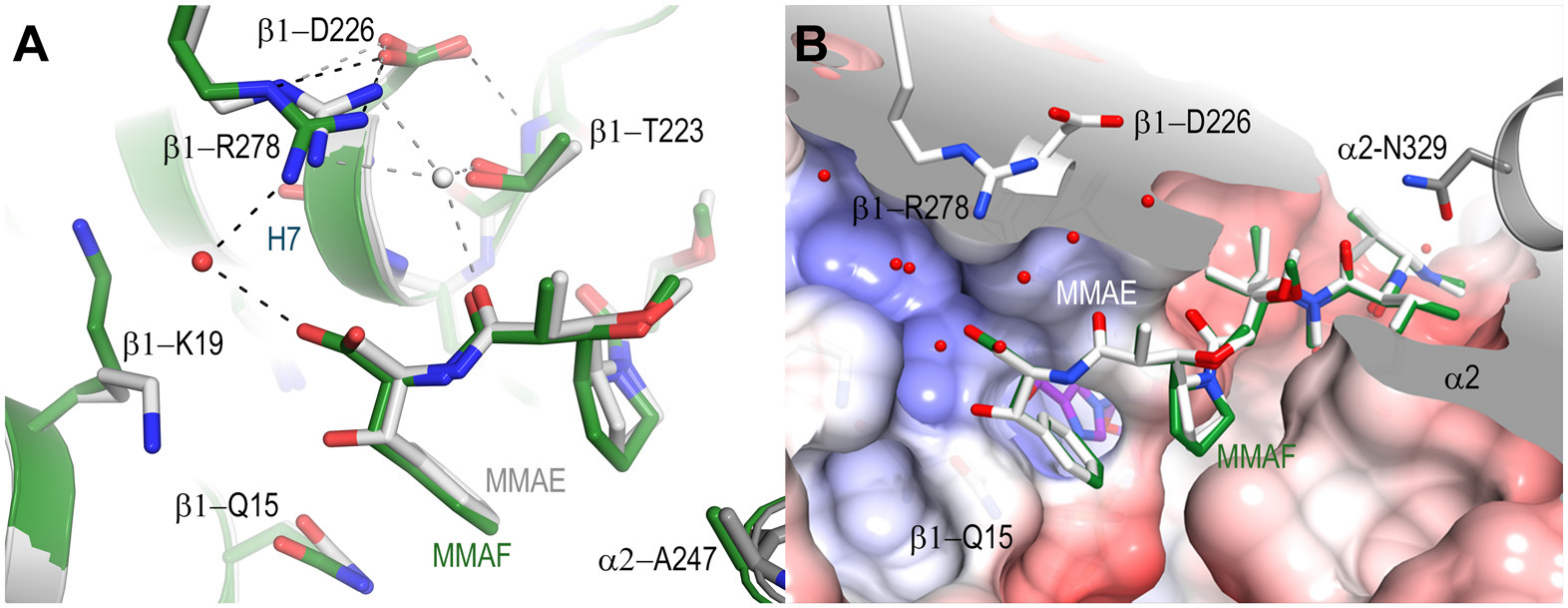
Comparison of the T_2_R-TTL-MMAF and the T_2_R-TTL-MMAE crystal structures. (**A**) Superposition of the MMAE structure (white) onto the MMAF structure (dark green) highlighting the subtle differences observed at the carboxy-terminal ends in the binding site. Hydrogen bonds and waters present in the MMAF structure are represented as black dashed lines and red spheres, those of the superimposed MMAE structure are in grey. The negatively charged carboxylate of MMAF interacts with the guanidinium group of Arg278 through a crystallographic water molecule. In the MMAE structure, the carbonyl group of Dap coordinates a crystallographic water molecule located in a central position to a complex network of hydrogen bonding interactions between Arg278, Asp226 and Thr223. Both these interactions may stabilize the interaction between Arg278 and Asp226 on βH7. (**B**) Surface sliced view of the MMAE binding site. The surface is colored by electrostatic potential from red (-8 KbT / ec) to blue (+8 KbT / ec). The positively charged amino-terminus interacts with the relatively negative charged region of the peptide binding site. The valine and Dil sidechains orient the MMAE through interactions with hydrophobic pockets on the β_1_ and α_2_ subunits. The carboxy-terminal ends of both the MMAE and MMAF molecules are solvent exposed and the interactions with the backbone amides of the βH6-H7 loop orient their carboxy-terminal groups in a positively charged and solvent filled pocket above the bound nucleotide.

Compared to MMAE, the specific interactions for the MMAF ligand are maintained at all positions with the exception of the carboxy-terminal phenylalanine group (Fig 3A). Although the phenyl group similarly occupies the groove between β_1_-tubulin Tyr224 and Gln15, there are no specific interactions with either Gln15 or a coordinated water molecule with Asn228 due to the lack of a hydroxyl in this position (Fig 3A). Despite the absence of a specific interaction, the Gln15 is still repositioned away from the bound GDP, suggesting that this change in the β_1_-tubulin subunit upon ligand binding is the result of steric interactions at the apical region of the nucleotide binding site. The carboxylic acid of the phenylalanine tilts towards the positively charged pocket defined by β_1_-tubulin His229, Lys19 and Arg278 (Fig 3B) and in this orientation a carboxylate oxygen coordinates a crystallographic water molecule which is shared via hydrogen bond to the guanidinium group of Arg278.

### Crystal structure of T_2_R-TTL in complex with vinblastine

**Fig 4:**
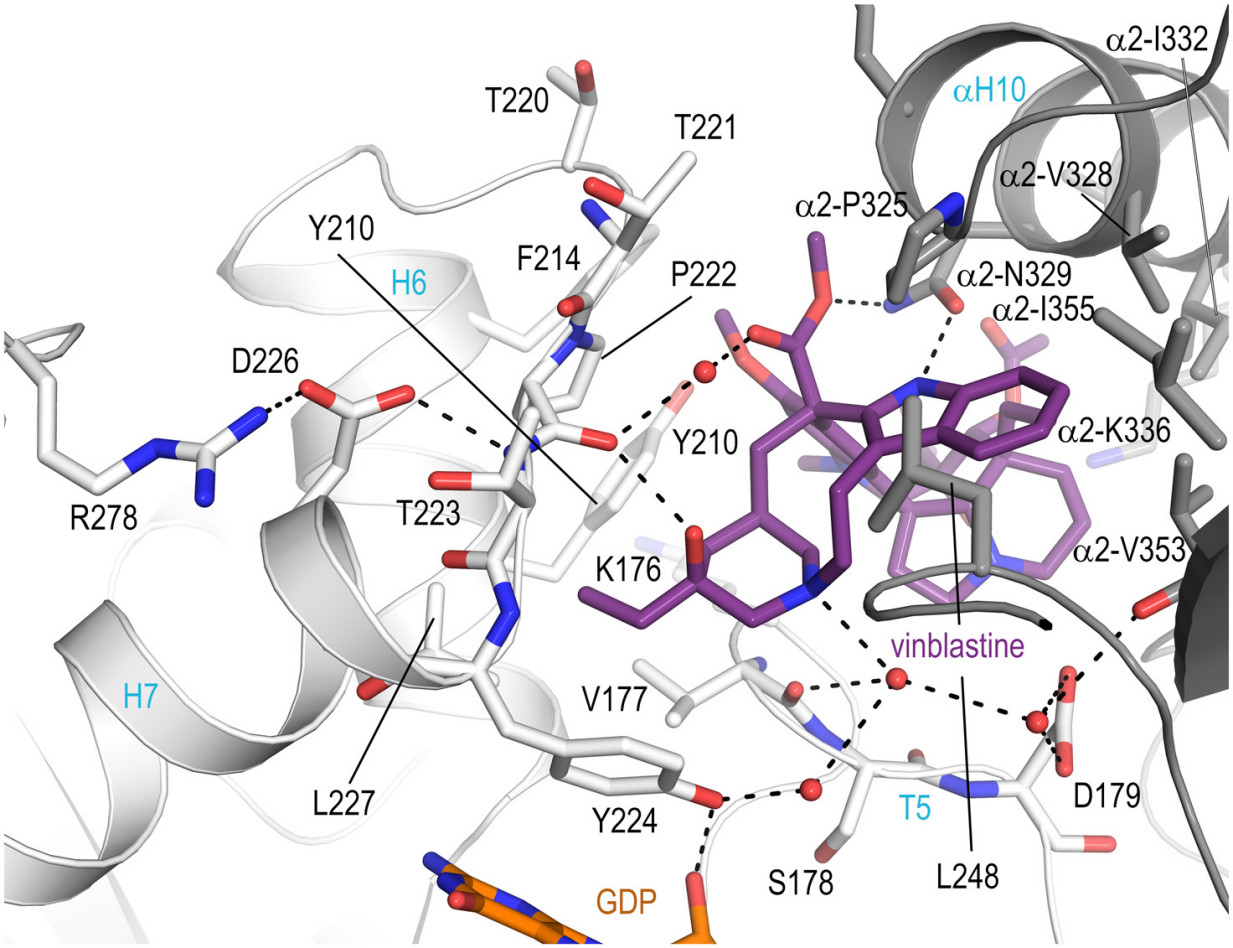
The detailed tubulin-vinblastine interactions at the vinca site. Vinblastine is in violetpurple stick representation. β_1_- and α_2_-tubulin are displayed as light and dark grey ribbons, respectively. Key residues forming the interaction with the ligand are in stick representation and are labeled. Hydrogen bonds are highlighted as dashed black lines, water molecules as red spheres.

The binding mode of vinblastine has been described in terms of tubulin secondary-structure elements due to the moderate resolution [15,16]. The high resolution crystal structure of the T_2_R-TTL-vinblastine complex presented in this study now allows to describe the detailed interactions in the binding site and to compare them to the binding of MMAE and MMAF. The vinblastine binding site at the interdimer interface is shaped by the β_1_–tubulin subunit T5 loop, the carboxy-terminal end of helix H6, the H6-H7 loop and the amino-terminus of the helix H7. The T7 loop, the S9 strand and the helix H10 of α_2_-tubulin shape the remaining part of the binding site (Fig 4). Similar to MMAE (Fig 2C) vinblastine forms two hydrogen bonds to Asn329, one direct and one water-mediated hydrogen bond to the backbone carbonyl of Pro222, thereby establishing a crosstalk to Asp226 and Arg278. Additional hydrogen bonds are formed by the tertiary amine of the catharanthine moiety to a water molecule connecting the T5 loop backbone carbonyl of Val177, the carboxylate of Asp179 and the hydroxyl group of Tyr224 that contacts the ribose 2’-hydroxyl group of the bound nucleotide (Fig 4).

### Extended M-loop stabilization by MMAs and vinblastine

**Fig 5:**
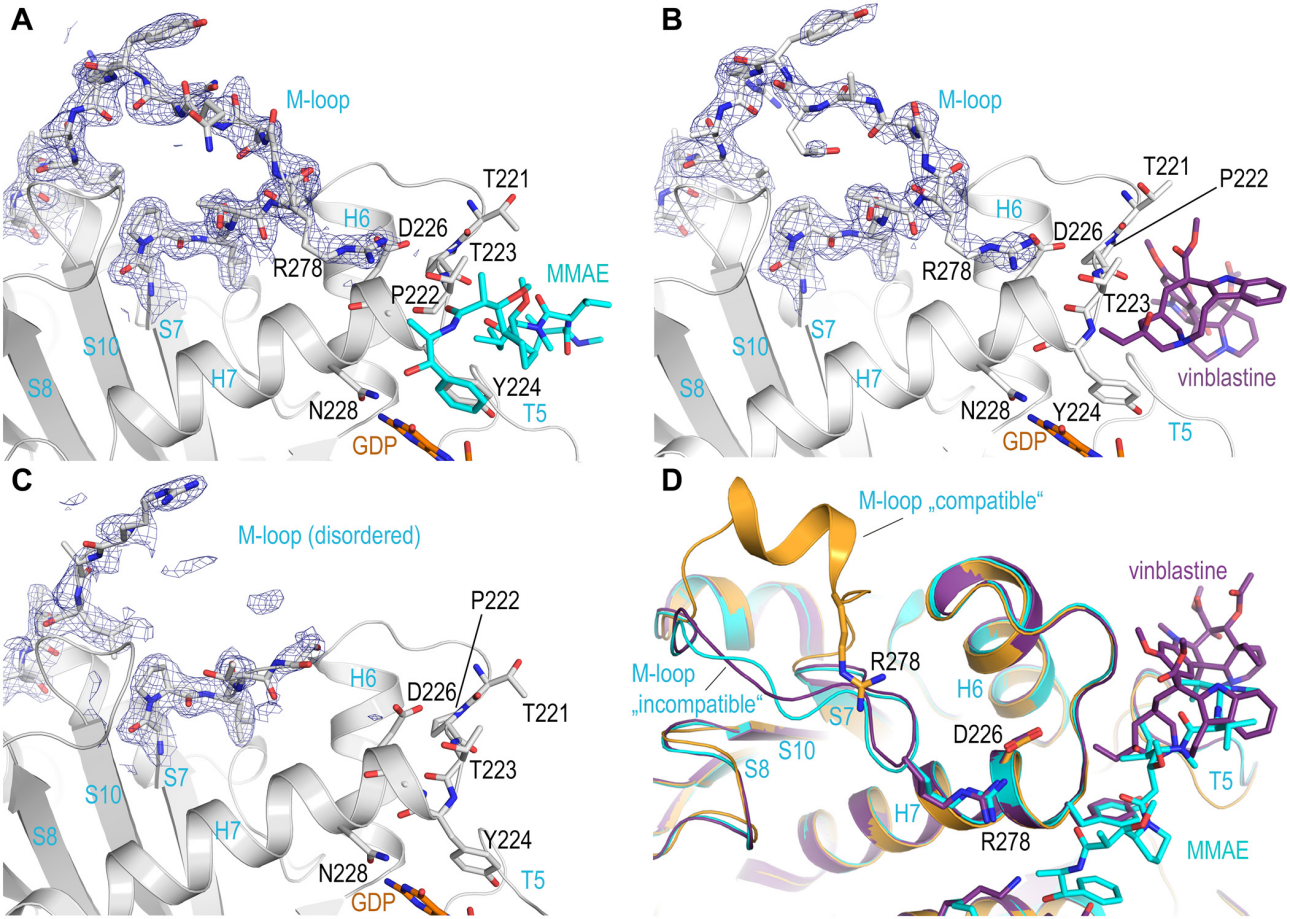
The effect of ligand binding on M-loop conformation. Electron density map details covering the M-loops of (**A**) the T_2_R-TTL-MMAE, (**B**) the T_2_R-TTL-vinblastine and (**C**) the T_2_R-TTL-apo crystal structures. The electron density maps (blue mesh) are contoured at 1.0 σ. In both the MMAE and the vinblastine complexes, the Arg278 interaction maintains the M-loop in an extended conformation incompatible with lateral contact formation in intact microtubules. (**D**) Ribbon representation of the superimposed T_2_R-TTL-MMAE (cyan), T_2_R-TTL-vinblastine (violetpurple) and the T_2_R-TTL-zampanolide complex (orange, PDB ID 4I4T). The ligands and the highlighted residues are represented as colored sticks and are labeled. Compared to the M-loop of the T_2_R-TTL-zampanolide complex, which adopts a conformation that is compatible with lateral protofilament contacts, both the M-loops of the liganded MMAE and vinblastine structures adopt a more extended conformation allowing Arg278 to interact with Asp226 and through water molecules with the carboxy-terminal portions of the MMA-ligands bound to the peptide site, thereby locking the M-loop in a incompatible conformation for lateral protofilament contacts.

The comparison of the three structures additionally highlights the key Arg278 residue on the β_1_-tubulin subunit that is in contact with MMAF via an ordered water molecule and which likely explains the increased affinity observed for MMAF. This interaction is of special interest because Arg278 is located on the highly flexible β-tubulin M-loop, a secondary structure element that becomes helically structured upon tubulin assembly into microtubules, and which partially defines the taxane site to which microtubule-stabilizing agents bind [27]. High resolution crystal structures of tubulin liganded with taxane-site binding agents show a well ordered helical M-loop region [21]. To date, crystal structures solved in the absence of such stabilizing agents exhibit an M-loop that is either disordered (T_2_R-TTL-apo, PDB ID 4IHJ, Fig 5C) or it preferentially adopts a poorly defined, extended conformation without clear secondary structure [15,18,22,28–34]. In this extended M-loop conformation the Arg278 side chain is favorably oriented to interact with Asp226 located at the amino-terminus of helix H7. This same orientation is observed in the T_2_R-TTL-MMAE, the T_2_R-TTL-MMAF and the T_2_R-TTL-vinblastine structures (Fig 5A and 5B), which either stabilize the ordered solvent surrounding the Arg278/Asp226 interaction through a distinct hydrogen bonding network (Fig 3A), or through the stabilization of the β_1_-tubulin H6/H7 loop (Fig 4). In the case of MMAF, the stabilization effect is even strengthened by the presence of a terminal carboxylate in proximity of the guanidinium group of Arg278. In the MMAE bound structure, the carboxylate is not present; however, an ordered crystallographic water molecule forms a bidentate interaction with Arg278 and is within hydrogen bonding distance to both Thr223 and the carbonyl of Dolaproine (Fig 3A). This extended hydrogen bond network to Arg278 may well serve to maintain the β-tubulin M-loop in an extended conformation that would be incompatible with the helical one seen in microtubules [35,36]. This conformation is distinct from the compatible conformation observed in the crystal structures of tubulin liganded by taxane site microtubule stabilizing agents (Fig 5D) and could therefore augment the destabilizing potential of auristatins.

## Conclusions

Our high-resolution crystallographic comparison of the binding modalities of MMAE and MMAF lends valuable insight into the specific interactions that give rise to the nano- and picomolar potency of these antimitotic agents in cellular assays. The analysis provides a structural explanation why the presence of a negatively charged phenylalanine group at the carboxy-terminus of MMAF increases the binding to free tubulin over the uncharged norephedrine of MMAE, and likely explains the >100fold increase in cytotoxicity exhibited by MMAF analogs [25]. We show that detailed structural interactions that extend the vinca domain to the peptide site are not only functionally distinct by inhibiting nucleotide exchange, but also indicate how peptide site antimitotics attain increased potency over the vinca alkaloids.

While preparing this manuscript, an article by Wang Y. and coworkers was published, which describes the crystal structures of both MMAE and vinblastine at 2.5 Å resolution [26]. The article by Wang and coworkers describes the binding modes of the ligands and discusses the pharmacophoric aspects of ligand binding to the vinca-site, which is distinct from the focus of the work described in this manuscript.

The established mechanisms of microtubule depolymerization by the auristatins include curvature induction, longitudinal polymerization and inhibition of nucleotide exchange. Our analysis of high resolution crystallographic structures now suggests the possibility of a fourth component, that of extended M-loop stabilization, to this highly potent class of antimitotics. Although additional research will be necessary to further explore this hypothesis, these structural findings represent a significant step towards our understanding of the cytotoxic potency of auristatins and other peptide-site binding agents at the atomic level.
